# L-carnitine suppresses transient receptor potential vanilloid type 1 activity and myofibroblast transdifferentiation in human corneal keratocytes

**DOI:** 10.1038/s41374-021-00538-0

**Published:** 2021-02-26

**Authors:** Elizabeth Turan, Monika Valtink, Peter S. Reinach, Annett Skupin, Huan Luo, Tobias Brockmann, Marah Hussain Omar Ba Salem, Uwe Pleyer, Stefan Mergler

**Affiliations:** 1 Klinik für Augenheilkunde, Charité—Universitätsmedizin Berlin, corporate member of Freie Universität Berlin, Humboldt-Universität zu Berlin and Berlin Institute of Health, Berlin, Germany; 2 Institute of Anatomy, Faculty of Medicine Carl Gustav Carus of the TU Dresden, Dresden, Germany; 3 School of Ophthalmology and Optometry, Wenzhou Medical University, Wenzhou, PR China; 4 Berlin Institute of Health (BIH), Kapelle-Ufer 2, 10117 Berlin, Germany; 5 Department of Ophthalmology, Universitätsmedizin Rostock, Rostock, Germany

**Keywords:** Ion channels, Diseases

## Abstract

Corneal stromal wound healing is a well-balanced process promoted by overlapping phases including keratocyte proliferation, inflammatory-related events, and tissue remodeling. L-carnitine as a natural antioxidant has shown potential to reduce stromal fibrosis, yet the underlying pathway is still unknown. Since transient receptor potential vanilloid 1 (TRPV1) is a potential drug target for improving the outcome of inflammatory/fibrogenic wound healing, we investigated if L-carnitine can mediate inhibition of the fibrotic response through suppression of TRPV1 activation in human corneal keratocytes (HCK). We determined TRPV1-induced intracellular calcium transients using fluorescence calcium imaging, channel currents by planar patch-clamping, and cell migration by scratch assay for wound healing. The potential L-carnitine effect on TRPV1-induced myofibroblast transdifferentiation was evaluated by immunocytochemical detection of alpha smooth muscle actin. RT-PCR analysis confirmed TRPV1 mRNA expression in HCK. L-carnitine (1 mmol/l) inhibited either capsaicin (CAP) (10 µmol/l), hypertonic stress (450 mOsmol/l), or thermal increase (>43 °C) induced Ca^2+^ transients and corresponding increases in TRPV1-induced inward and outward whole-cell currents. This was accompanied by suppression of injury-induced increases in myofibroblast transdifferentiation and cell migration. In conclusion, L-carnitine contributes to inhibit stromal scarring through suppressing an injury-induced intrinsic TRPV1 activity that is linked with induction of myofibroblast transdifferentiation in HCK cells.

## Abstract (web summary)

Human corneal keratocytes (HCK) are essential for maintaining corneal structure and transparency. This study shows that the natural antioxidant L-carnitine inhibits stromal scarring by suppressing injury-induced intrinsic transient receptor potential vanilloid type 1 (TRPV1) activity that is linked to induction of myofibroblast transdifferentiation in HCK cells. Blocking TRPV1 activation on keratocytes may therefore be a viable approach to suppress corneal opacification in a clinical setting.

## Introduction

Human corneal keratocytes (HCK) or fibroblasts are interspersed between orthogonally arranged layers of collagen lamellae in the stroma. They are essential for maintaining corneal structure and transparency as they express stromal collagen fibers and different constituents of the extracellular matrix [1]. Under a quiescent condition, HCK elaborate constituents contributing to the formation of an organized collagenous transparent framework stroma, but during exposure to various stresses or infection HCK can be induced to undergo transdifferentiation into fibroblasts and myofibroblasts. This transition is essential for inducing the responses underlying wound repair and restoration of normal visual function. However, if this response is dysfunctional as a consequence of excessive fibroblast activation and transdifferentiation into myofibroblasts, the cornea can undergo opacification due to excessive irreversible scarification [2].

Injury-induced corneal scarring fibrosis and opacification are mediated through TRPV1 activation upregulating TGFβ-expression which in turn promotes keratocyte transdifferentiation into myofibroblasts and increases in smooth muscle α-actin expression [3]. This finding in concert with the identification of functional expression of TRPV1 on fibroblasts help explain why this non-selective ion channel is a recognized target to suppress myofibroblast transdifferentiation and corneal scarring. Such an outcome is expected to improve restoration of tissue transparency as a consequence of wound healing induced by severe injury or infection [4, 5]. On the other hand, there is a caveat to this approach since epithelial TRPV1 activation instead promotes wound closure of this tissue. In order to circumvent this complication, intrastromal injection of an antagonist may be an option if it only blocks TRPV1 on stromal keratocytes and fibroblasts [6].

Osmoprotective agents have also been identified which lessen injury-induced stimulation of signaling pathways promoting tissue scarring during wound healing. Carnitine is one such agent (β-hydroxy-γ-N-trimethylaminobutyric acid) [7]. It is a nutritional supplement widely distributed in foods of animal origin. A protective effect of L-carnitine was described in retinal pigment epithelial cells [8] and human corneal epithelial cells (HCEC) [9, 10]. In conjunctival epithelial cells (HCjEC), L-carnitine reduced hypertonic-induced cell shrinkage through interacting with TRPV1 channels [11]. L-carnitine also functions as an osmoprotectant to suppress inflammatory responses via inhibiting TRPV1 linked signaling pathway in hyperosmotically stressed HCEC [9]. Finally, L-carnitine reduced oxidative stress-induced upregulation and stimulation of matrix metalloproteinase (MPs) activity resulting from exposure to hyperosmolarity in HCEC [10, 12]. These results prompted us to determine if L-carnitine application also reduces corneal stromal responses underlying opacification through inhibiting TRPV1 activation on HCK.

We show here that L-carnitine suppresses TRPV1 activation induced by exposure to either capsaicin (CAP), elevated temperature, or hypertonic stress in HCK. In addition, L-carnitine suppressed injury-induced increases in HCK migration. Its potential usefulness as a therapeutic agent was demonstrated by showing that it inhibited TRPV1-induced keratocyte transdifferentiation into myofibroblasts. This result is relevant since it is known that this transition underlies corneal scarring and opacification, which can be symptomatic in patients chronically afflicted with stromal infection.

## Materials and methods

### Cell culture of HCK

SV40-immortalized HCK cells derived from a human cornea were used as a representative cell model of corneal keratocytes [13–15]. HCK identity was validated based on confirming specific keratocyte biomarker expression [4]. Cells were grown in DMEM medium containing 10% fetal calf serum as well as penicillin/streptomycin in a humidified 5% CO_2_ incubator at 37 °C. For the electrophysiological measurements, cell confluence ranged between 60 and 80%. Over this range of confluence, their electrophysiological characteristics were invariant [4].

### RNA isolation, RT-PCR, and sequencing

HCK cells were seeded in T25 flasks and grown to confluence. Total RNA was extracted using TriFast^TM^ (PeqLab, by VWR, Darmstadt, Germany) according to the manufacturer’s instructions. RNA concentration and purity were determined with a NanoPhotometer™ (Implen GmbH, Munich, Germany) and it was stored at −80 °C until further use. RNA (1.8 µg per sample) was reverse transcribed with RevertAid H minus first strand cDNA synthesis kit (Thermo Fisher, Waltham, USA) according to the manufacturer´s instructions in a total volume of 20 µl, and cDNA was stored at −80 °C until further use. PCR was performed with a 2 µl cDNA mixture in a total volume of 35 µl using Taq polymerase and 0.2 nmol/µl of each forward and reverse primer (intron spanning, 5′‑CCCCCGATAGCTCCTACAAC‑3′ and 3′‑AAGGCCTTCCTCATGCACT‑5′, TRPV1 mRNA accession number NM_018727) after initial 95 °C for 3 min over 35 cycles of 95 °C for 30 s, 64 °C for 30 s and 72 °C for 45 s, finalized by 72 °C for 7 min and a temperature hold at 4 °C in a Techne TC-512 gradient thermal cycler (Staffordshire, UK), yielding a 295 bp product. Samples with RNAse- and DNAse-free H_2_O instead of cDNA template and samples that were proceeded without reverse transcriptase in the reverse transcription reaction served as non-template controls. Total RNA from a confluent human corneal endothelial cell line (HCEC-12) served as positive control [16]. PCR products were electrophoresed on a 1.5% agarose gel at 120 V for 1.75 h and visualized with ethidium bromide. Bands were cut out and DNA was eluted from the agarose using MP Biomedicals™ Geneclean™ Turbo Kit (Thermo Fisher) in a total elute volume of 30 µl. Eluted PCR products were sequenced (Eurofins Genomics GmbH, Ebersberg, Germany) and sequencing results were checked with NCBI Blast® software (https://blast.ncbi.nlm.nih.gov).

### Fluorescence calcium imaging

HCK cells were pre-incubated with culture medium containing 1 µmol/l fura-2/AM for ≈40 min. The loading was stopped with a Ringer-like (control) solution containing (mmol/l): 150 NaCl, 6 CsCl, 1 MgCl_2_, 10 glucose, 10 HEPES, and 1.5 CaCl_2_ at pH 7.4 and 317 mOsmol/l [17]. At room temperature (≈23 °C), intracellular free Ca^2+^ ([Ca^2+^]_i_) levels were measured based on fura-2 emission at 510 nm resulting from alternating excitation in a 5 s loop at 340 and 380 nm with a digital imaging system (Olympus Europa Holding GmbH, Hamburg, Germany). Cells were cultured on coverslips (diameter 15 mm) and put in a chamber containing the aforementioned Ringer-like solution. Single cells were selected and designated as regions of interest. Cells were exposed to UV-light within a 5-s interval using a LED light source (LED-Hub by Omikron, Rodgau-Dudenhofen, Germany). The images were simultaneously recorded with a digital camera (Olympus XM-10) and *f*_340nm_/*f*_380nm_ fluorescence ratios, which are proportional to [Ca^2+^]_i_, were calculated by cellSens software (Olympus Europa Holding GmbH, Hamburg, Germany). Fluorescence ratios were normalized (control set to 0.1) and averaged (with error bars) and results shown as mean traces of the *f*_340nm_/*f*_380nm_ ratio ± SEM (error bars in both directions) with n*-*values indicating the number of experiments per data point. Ca^2+^ increases were time delayed because drugs were pipetted into a stationary bath rather than a flow through system. L-carnitine effects were evaluated following a 30 min preincubation period. Drug stock solutions were prepared with dimethyl sulfoxide (DMSO) and diluted to obtain a nontoxic working concentration wherein DMSO did not exceed 0.1%. Hypertonic stress (i.e., 450 mOsmol/l) was imposed by supplementing isotonic Ringer-like solution with 130 mmol/l D-mannitol to obtain a hypertonic Ringer-like solution.

### Scratch wound cell migration assay

HCKs were incubated in 12-well plates for 48 h until the cells reached confluence, which was then scratched using a pipette tip. The cell layer was subsequently washed twice with PBS, and then fresh medium was added. The non-treated cells served as controls whereas those treated with 1 mmol/l L-carnitine were incubated for either another 8 or 24 h. Images were acquired at 0, 8, and 24 h post treatment using phase-contrast microscopy with a ×10 objective lens. The open wound areas were calculated using Image software (3 replicates).

### Immunocytochemistry

HCK cells were cultured for 3 days in 12-well cell culture plates. Before fixation, HCK cells were stimulated for 24 h with 5 ng/ml human recombinant transforming growth factor beta 1 (TGFβ1, ab50036, Abcam, Cambrigde, United Kingdom), 10 µmol/l capsazepine (CPZ), 1 mmol/l L-carnitine, 5 ng/ml TGFβ1 plus 10 µmol/l CPZ, whereas 5 ng/ml TGFβ1 plus 1 mmol/l L-carnitine with Dulbecco’s Modified Eagle Medium (DMEM) served as a control. Vital cells were fixed for 30 min using 4% formaldehyde, followed by washing three times with Tris-buffered saline (TBS; pH 7.6, 10 min each). Cells were permeabilized with 0.1% Triton-X100 (15 min) and blocked with 5% BSA in TBS for 60 min. HCK cells were incubated overnight in a humidified chamber at 4 °C with primary alpha-smooth muscle actin (αSMA) antibodies (1:200, 701457, Invitrogen, Carlsbad, California, USA) diluted in 0.8% BSA in TBS. Fluorescence detection employed Cy3 conjugated secondary antibodies (1:200, C2306; Sigma-Aldrich, St. Louis, Missouri, USA) and DAPI nuclear counterstaining. Mounted slides were examined using light microscopy (Axio Imager.M2; Zeiss, Jena, Germany).

### Planar patch-clamp recordings

HCK whole cell currents were evaluated as previously described [4]. In brief, a standard intracellular solution containing (mmol/l): 50 CsCl, 10 NaCl, 60 CsF, 20 EGTA, and 10 HEPES at pH ≈ 7.2 and ≈288 mOsmol/l was applied to the microchip (both provided by Nanion, Munich, Germany). The external solution contained (mmol/l): 140 NaCl, 4 KCl, 1 MgCl_2_, 2 CaCl_2_, 5 D-glucose monohydrate, and 10 HEPES, pH ≈ 7.4 and osmolarity ≈ 298 mOsmol/l. Mean membrane capacitance (17 ± 6 pF; *n* = 7) and mean access resistance (7 ± 2 MΩ; *n* = 7) were calculated by the software. Series resistances, fast and slow capacitance transients were compensated by the amplifier in conjunction with the PatchMaster software. Current recordings were all leak-subtracted and cells with leak currents above 100 pA were discarded. Cells were depolarized every 5 s from −60 to +130 mV in 500 ms steps (voltage ramp). The holding potential (HP) was set to 0 mV in order to eliminate any possible contribution by voltage-dependent Ca^2+^ channel activity. Experiments were started ~10 min after breaking into a whole-cell configuration [18]. Resulting currents were normalized using cell membrane capacitance to obtain current density (pA/pF).

### Statistical analysis

Parametric Student’s *t*-test for paired and unpaired data was used to determine if the data passed the normality test. Otherwise, the nonparametric Wilcoxon test was used. *P* values < 0.05 were considered as significant. All values in the bar charts are shown as means ± SEM (error bars in both directions). Significance was determined using the GraphPad Prism software (version 5.00 for Windows) (La Jolla, California, USA). The bar charts were generated with the same software. All other plots were generated with the SigmaPlot software version 12.5 for Windows (Systat Software, San Jose, California, USA).

## Results

### TRPV1 expression and function in HCK

**Fig. 1:**
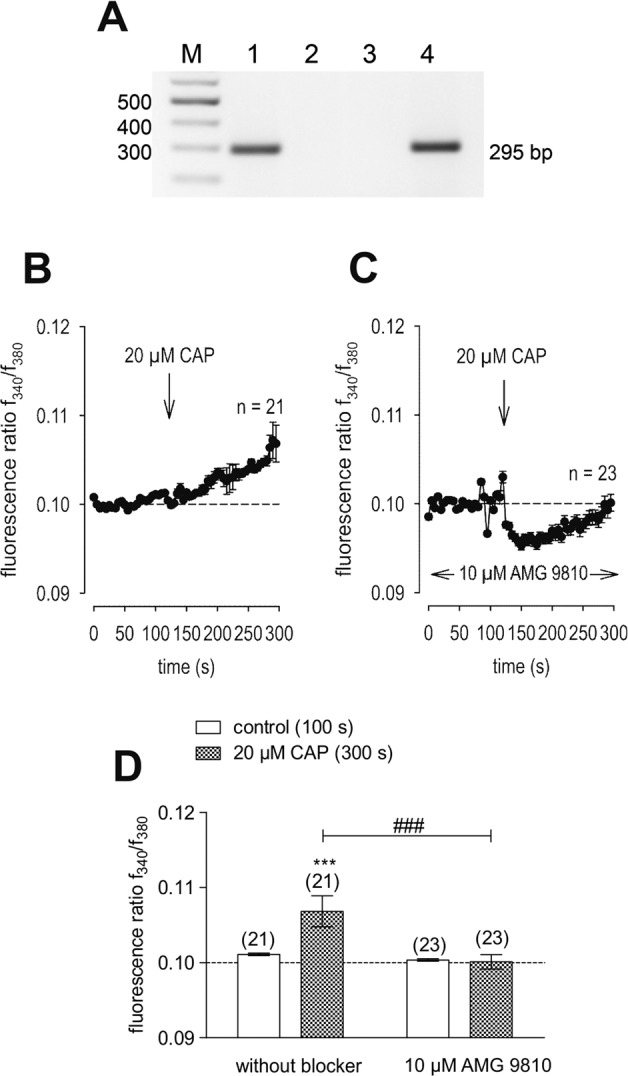
TRPV1 gene and functional expression in HCK. **A** RT-PCR analysis of TRPV1 expression using gene specific primers revealed TRPV1 in HCK (lane 1) and HCEC-12 (positive control, lane 4). Template-free negative controls (lane 2: template replaced with H_2_O, lane 3: w/o reverse transcriptase in the reverse transcription reaction) gave no bands. *M* = 100 bp plus DNA ladder. **B** 20 µmol/l CAP induced an increase in Ca^2+^ influx (*n* = 21) in HCK. **C** Same experiment as shown in **B**, but in the presence of AMG 9810. AMG 9810 (10 µmol/l) clearly suppressed the CAP-induced Ca^2+^ increase even partially below the base line level (*n* = 23). **D** Summary of the experiments with CAP and AMG 9810 in HCK. The asterisks (*) designate significant increases in [Ca^2+^]_i_ with CAP (*t* = 300 s; *n* = 21; p < 0.005; paired tested) compared to control (*t* = 100 s). The hashtags (#) indicate statistically significant differences in fluorescence ratios between CAP with and without AMG 9810 (*t* = 300 s; *n* = 21–23; *p* < 0.005; nonpaired tested).

Figure 1A provides a result consistent with TRPV1 gene expression based on obtaining the predicted 295 bp RT-PCR product with intron-spanning specific primers. Its identity was confirmed based on the amplicon sequencing result (data not shown). Negative signals in the non-template control reactions as well as those obtained with intron-spanning primers confirmed specific amplification of only cDNA and excluded primer binding to genomic DNA. HCEC-12 were used as a positive control since TRPV1 gene expression was documented in earlier studies [16]. In addition, Fig. 1B–D confirm functional TRPV1 expression based on using CAP and the specific TRPV1 blocker AMG 9810 [19]. Specifically, extracellular application of 20 µmol/l CAP increased the fluorescence ratio (*f*_340_/*f*_380_) from 0.1011 (100 s) ± 0.0002 to 0.1068 ± 0.0021 after 300 s (*n* = 21; *p* < 0.005; Fig. 1B, D). In the presence of 10 µmol/l AMG 9810, this CAP-induced Ca^2+^ increase could be clearly suppressed to 0.1001 ± 0.0010 at 300 s (*n* = 23; *p* < 0.005; Fig. 1C, D).

**Fig. 2:**
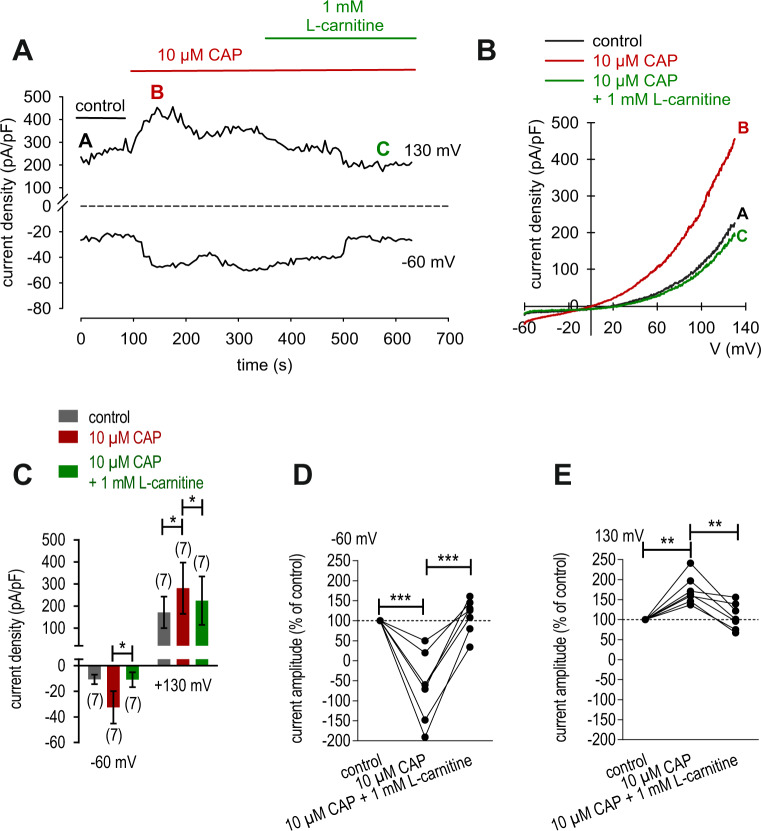
L-carnitine suppressed CAP-induced increases of whole-cell currents in HCK. **A** Time course recording of the current increases induced by CAP (10 µmol/l) and declined after application of 1 mmol/l L-carnitine. **B** Original traces of CAP-induced current responses to voltage ramps. Current densities are shown before application (labeled as **A**), during application of 10 µmol/l CAP (labeled as **B**) and after addition of 1 mmol/l L-carnitine (labeled as **C**). Calculated current densities obtained by normalizing currents to membrane capacitance as function of imposed voltage were derived from the traces shown in **A**. **C** Summary of patch-clamp experiments with CAP and L-carnitine. The asterisks (*) indicate statistically significant differences of whole-cell currents with and without CAP (*n* = 7; *p* < 0.05; paired tested) and significant difference of CAP-induced increased with and without L-carnitine (*n* = 7; *p* < 0.05; paired tested). **D** Maximal negative current amplitudes induced by a voltage step from 0 to −60 mV are depicted in percent of control values before application of 10  l/l CAP (control set to 100%). CAP-induced inward currents could be clearly suppressed in the presence of 1 mmol/l L-carnitine. **E** Same diagram but related to maximal positive current amplitudes induced by a voltage step from 0 to +130 mV.

Whole cell patch-clamp recordings are also supportive of functional TRPV1 expression in HCK since 10 µmol/l CAP induced increases in whole cell currents (Fig. 2). These increases were evaluated based on the plots of the corresponding current voltage relationships at the time points designated as: A, B and C (Fig. 2A, B). At positive potentials, this TRPV1 agonist increased the typical outward TRPV1-like rectifying currents from 172 ± 71 pA/pF to 281 ± 117 pA/pF (*n* = 7; *p* < 0.05) (*n* = 7; *p* < 0.05) (Fig. 2B, C). Similarly, maximal inward current amplitudes induced by a voltage step from 0 to −60 mV increased to −66 ± 32% of control (*n* = 7; *p* < 0.005) (Fig. 2D). Maximal outward current amplitudes induced by a voltage step from 0 to +130 mV increased to 173 ± 13% of control (control set to 100%) (*n* = 7; *p* < 0.01) (Fig. 2E).

### L-carnitine inhibits TRPV1 activation

**Fig. 3:**
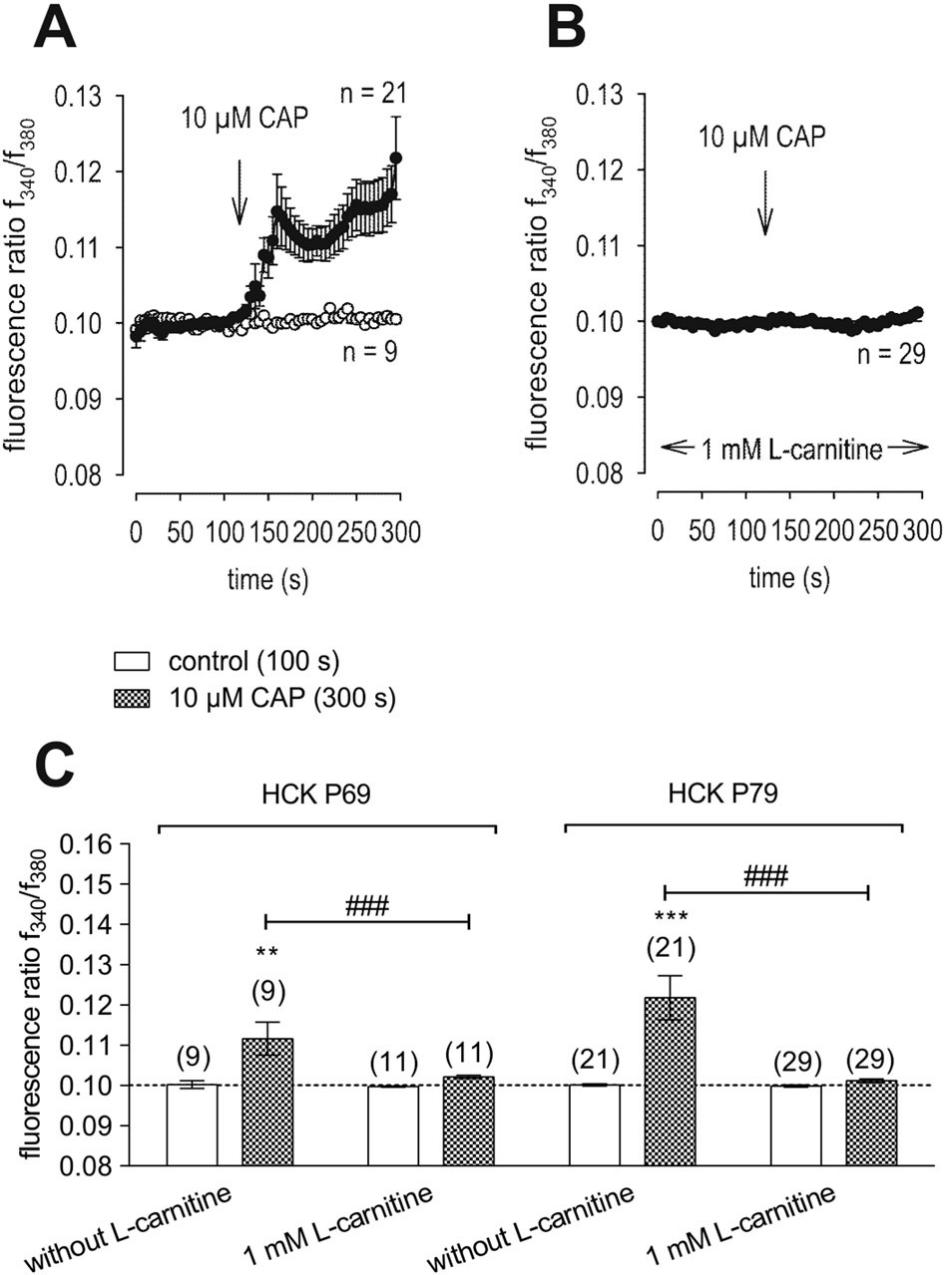
L-carnitine suppressed CAP-induced increases of intracellular Ca^2+^ concentration. **A** 10 µmol/l CAP induced an increase in Ca^2+^ influx (*n* = 21) in HCK (P79) whereas non-treated control cells maintained a constant Ca^2+^ baseline (*n* = 9). **B** Same experiment as shown in **A**, but in the presence of L-carnitine (HCK P79). L-carnitine (1 mmol/l) clearly suppressed the CAP-induced Ca^2+^ increase (*n* = 29). **C** Summary of the experiments with CAP and L-carnitine in HCK from two different cell passages (P69, P79). The asterisks (*) designate significant increases in [Ca^2+^]_i_ with CAP (*t* = 300 s; *n* = 9–21; *p* < 0.01 at the minimum; paired tested) compared to control (*t* = 100 s). The hashtags (#) indicate statistically significant differences in fluorescence ratios between CAP with and without L-carnitine (*t* = 300 s; *n* = 9–29; *p* < 0.005; non-paired tested).

**Fig. 4:**
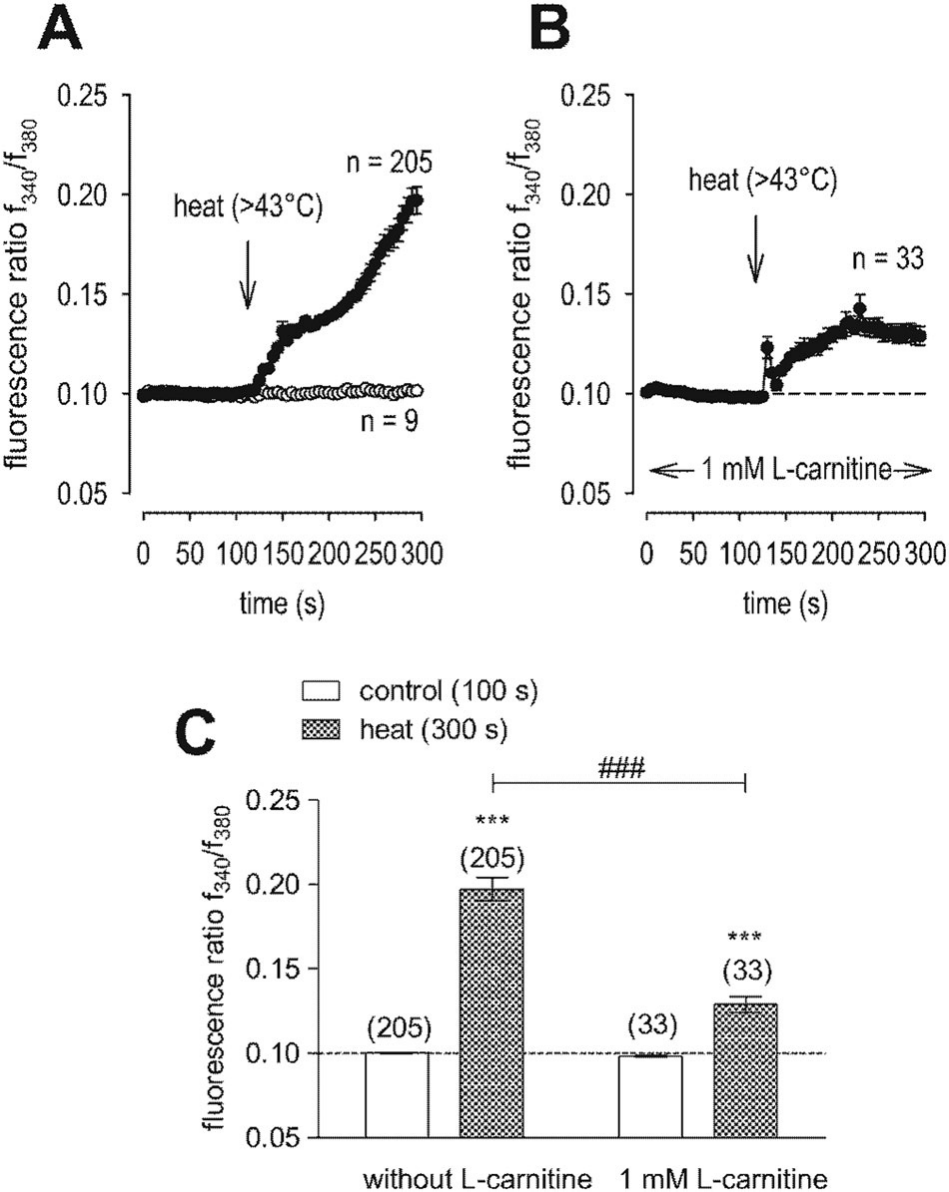
L-carnitine suppressed heat-induced increases of intracellular Ca^2+^ concentration. **A** Heat (>43 °C) induced an increase in Ca^2+^ influx (*n* = 205) whereas non-treated control cells maintained a constant Ca^2+^ baseline (*n* = 9). **B** Same experiment as shown in **A**, but in the presence of L-carnitine. L-carnitine (1 mmol/l) partially suppressed the heat-induced Ca^2+^ increase (*n* = 33). **C** Summary of the experiments with heat and L-carnitine. The asterisks (*) designate significant increases in [Ca^2+^]_i_ with heat (*t* = 300 s; *n* = 205; *p* < 0.005; paired tested) compared to control (*t* = 100 s). The hashtags (#) indicate statistically significant differences in fluorescence ratios between heat with and without L-carnitine (*t* = 300 s; *n* = 33–205; *p* < 0.005; non-paired tested).

**Fig. 5:**
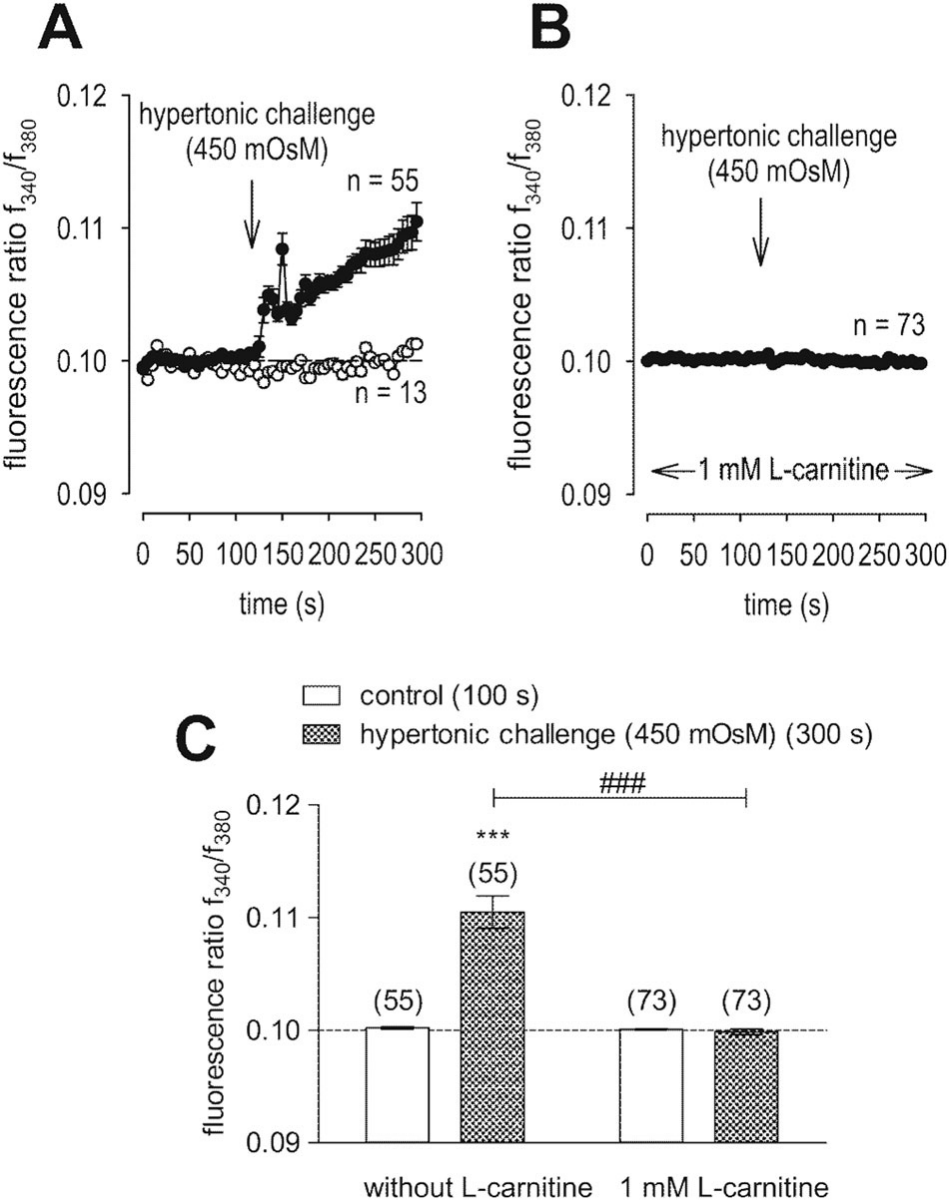
L-carnitine suppressed hypertonic stress-induced increases of intracellular Ca^2+^ concentration. **A** Hypertonic challenge (450 mOsmol/l) induced an increase in Ca^2+^ influx (*n* = 55) whereas non-treated control cells maintained a constant Ca^2+^ baseline (*n* = 13). **B** Same experiment as shown in **A**, but in the presence of L-carnitine. L-carnitine (1 mmol/l) clearly suppressed the hypertonic-induced Ca^2+^ increase (*n* = 73). **C** Summary of the experiments with hypertonicity and L-carnitine. The asterisks (*) designate significant increases in [Ca^2+^]_i_ with hypertonicity (*t* = 300 s; *n* = 55; *p* < 0.005; paired tested) compared to control (*t* = 100 s). The hashtags (#) indicate statistically significant differences in fluorescence ratios between hypertonic challenge with and without L-carnitine (*t* = 300 s; *n* = 55–73; *p* < 0.005; non-paired tested).

Figure 2 shows that L-carnitine (1 mmol/l) suppressed CAP-induced increases of whole-cell currents induced by a voltage step from −60 to +130 mV. CAP (10 µmol/l) increased both in- and outward currents, whereas 1 mmol/l L-carnitine suppressed them from −32 ± 13 pA/pF to −11 ± 6 pA/pF (*n* = 7; *p* < 0.05) (Fig. 2A–C) and outward current density was suppressed from 281 ± 117 pA/pF to 225 ± 109 pA/pF (both *n* = 7; *p* < 0.05) (Fig. 2C). Similarly, maximal inward current amplitudes induced by a voltage step from 0 to −60 mV decreased them to 112 ± 16% of control (*n* = 7; *p* < 0.005) (Fig. 2D) and maximal outward current amplitudes induced by a voltage step from 0 to +130 mV decreased to 108 ± 12% of control (*n* = 7; *p* < 0.01) (control set to 100%) (Fig. 2E). Similarly, extracellular application of 10 µmol/l CAP increased the fluorescence ratio (*f*_340_/*f*_380_) rose from 0.1001 (100 s) ± 0.0003 to 0.1218 ± 0.0055 after 300 s (*n* = 21; *p* < 0.005; Fig. 3A, C). With HCK cell passage 79, this increase was suppressed in the presence of 1 mmol/l L-carnitine (*f*_340_/*f*_380_ = 0.1011 ± 0.0004; *t* = 300 s; *n* = 29; *p* < 0.005) (Fig. 3B, C). Similar results were obtained using HCK at earlier cell passages (P69, Fig. 3C). Raising the bath temperature to >43 °C, which typically activates TRPV1 [20], increased the f_340_/f_380_ ratio from 0.1002 ± 0.0003 (100 s) to 0.1971 ± 0.0068 after 300 s (*n* = 205; *p* < 0.005; Fig. 4A, C). This increase fell to 0.1289 ± 0.0047 after 300 s when cells were preincubated with L-carnitine (*n* = 33; *p* < 0.005) (Fig. 4B, C). Hypertonicity irreversibly increased the fluorescence ratio from 0.1002 ± 0.0001 (100 s) to 0.1105 ± 0.0014 after 300 s (*n* = 55; *p* < 0.005; Fig. 5A, C). Following preexposure to 1 mmol/l L-carnitine in isotonic medium, the fluorescence ratio remained constant following substitution of 450 mOsmol/l Ringer-like solution containing L-carnitine (*n* = 73; *p* > 0.05) (Fig. 5B, C). In summary, these results confirm that 1 mmol/l L-carnitine acts as a TRPV1 antagonist in HCK cells.

### L-carnitine suppresses TRPV1-induced HCK migration

**Fig. 6:**
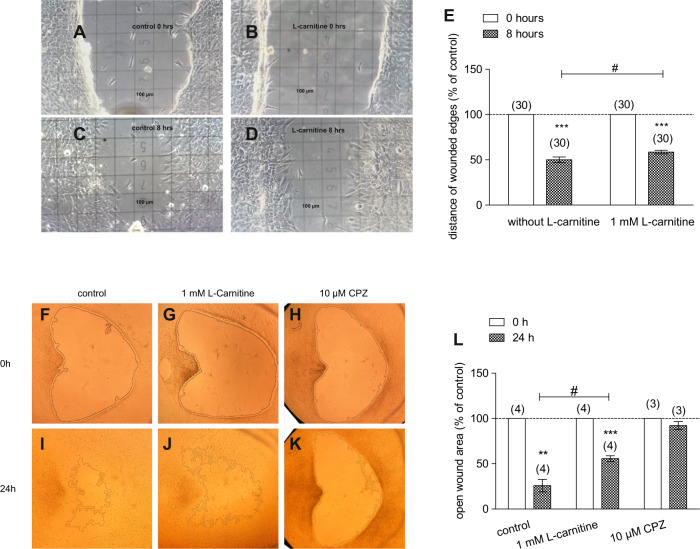
HCK migration with and without L-carnitine. When HCK reached confluence, a scratch was created with a pipette tip, and floating cells were removed by washing with PBS. The wounded edges and open wound areas were then observed from 0 to 8 and 24 h of treatment with and without the treatment of 1 mmol/l L-carnitine. Images of cell migration were acquired and microscopic fields of view of representative experiments are shown. **A** Light microscopic image of HCK at 0 h (control) (square = 100 µm). **B** HCK in the presence of 1 mmol/l L-carnitine. **C** HCK (control) after 8 h. **D** HCK (L-carnitine) after 8 h. **E** Summary of the experiment regarding distance of wounded edges (control set to 100%). The asterisks (*) designate significant decreases of the distances of the wounded edges (% of control) (*n* = 30; *p* < 0.005; paired tested) compared to control. The hashtag (#) indicates statistically significant difference of the wounded edges with and without the treatment of L-carnitine (*n* = 30; *p* < 0.05; non-paired tested). **F**–**K** Same experimental design as shown in **A**–**D**, but with focus on open wound area between 0 and 24 h. The open wound areas were marked by the software. Furthermore, CPZ was used as a negative control. **L** Summary of the experiments regarding open wound areas (control set to 100%). The asterisks (*) designate significant decreases of the open wound areas (% of control) (*n* = 3–4; *p* < 0.01–0.005; paired tested) compared to control. In contrast, the open wound areas did not decrease in the presence of CPZ. The hashtag (#) indicates statistically significant differences in open wound areas (% of control) between control and L-carnitine (*t* = 24 h; *n* = 4; *p* < 0.05, unpaired tested).

The effect of 1 mmol/l L-carnitine on HCK migration was calculated as a percentage based on the difference between the initial wound area at time point 0 h and the remaining wound area after 8 h in the presence and absence of carnitine. Figure 6A–E shows the two wound edges at 0 h in the control and with 1 mmol/l L-carnitine after 8 h. The distance separating the wound edges was larger with L-carnitine (*n* = 30; *p* < 0.05). After allowing HCK migration to proceed for 24 h, the area of the open wound was 30% larger than in the control (control = 26 ± 7%; 1 mmol/l L-carnitine = 56 ± 3%; *n* = 4; *p* < 0.05), indicating that L-carnitine inhibited wound closure (Fig. 6F–L). Moreover, the TRPV1 channel blocker capsazepine (CPZ) (10 µmol/l) as a positive control almost completely inhibited wound closure since the area of the open wound area was 66% larger than in the control (control = 26 ± 7%; 10 µmol/l CPZ = 92 ± 4%; *n* = 3–4; *p* < 0.005) (Fig. 6H, K, L). On the other hand, the open wound area in samples with 1 mmol/l L-carnitine was 15% smaller than that in samples with 10  µmol/l CPZ (Fig. 6L). This difference suggests that CPZ efficacy in inhibiting HCK migration is greater than that of L-carnitine.

### L-carnitine suppresses myofibroblast transdifferentiation

**Fig. 7:**
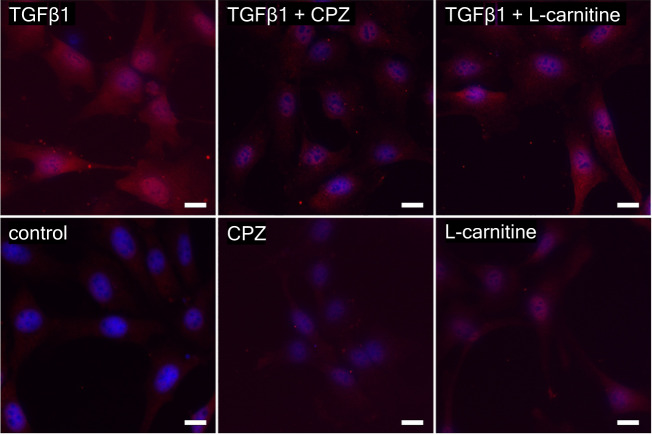
Myofibroblast activation in HCK after stimulations for 24 h with 5 ng/ml human recombinant transforming growth factor beta 1 (TGFβ1), 10 µmol/l capsazepine (CPZ), 1 mmol/l L-carnitine, 5 ng/ml TGFβ1 plus 10 µmol/l CPZ, 5 ng/ml TGFβ1 plus 1 mmol/l L-carnitine and, as a control, with Dulbecco’s Modified Eagle Medium (DMEM). Myofibroblasts become apparent as cells that stain positive for αSMA in the cytoplasm. Nuclear staining of HCK with DAPI (blue) and anti αSMA IF antibody red staining detecting myofibroblasts in HCK. Scale bar = 20 µm.

The functional contribution of TRPV1 in controlling the HCK transdifferentiation into myofibroblasts was evaluated by determining if L-carnitine inhibited TGFβ-induced keratocyte transdifferentiation into myofibroblasts [21]. This is a relevant approach since corneal injury leads to opacification as a consequence of TGFβ-induced TRPV1 upregulation followed by increases in keratocyte transdifferentiation into myofibroblasts. Accordingly, the ability of L-carnitine to suppress HCK transdifferentiation was investigated by immunostaining against the myofibroblast biomarker αSMA in the cytoplasm. Preincubation with 5 ng/ml TGFβ for 24 h increased αSMA-positive cytoplasmic staining, which is indicative of myofibroblast transdifferentiation (Fig. 7) [21]. In the untreated control cultures, cytoplasmic αSMA staining was absent. During exposure to either 10 µmol/l CPZ or 1 mmol/l L-carnitine, αSMA immunostaining was reduced to levels close to those in the untreated cultures. Specifically, in the presence of TGFβ1 plus L-carnitine, there was a slight reduction in αSMA-positive cytoplasmic staining, whereas TGFβ1 plus CPZ resulted in a more prominent reduction in cytoplasmic αSMA immunostaining. Nevertheless, the similarity between these two trends suggests that L-carnitine reduced TRPV1 activation by TGFβ.

## Discussion

### Functional TRPV1 expression

We document here gene and functional TRPV1 expression in an immortalized HCK cell line (Figs. 1, 2A, 3A) based in part on the effects of CAP and AMG 9810, which are validated TRPV1 channel modulators [19, 22]. Its presence agrees with similar previous studies in primary HCK cells and corneal fibroblasts [4, 5]. The electrophysiological responses that are signatures of TRPV1 activity such as outwardly rectifying currents, a reverse potential near 0 mV (Fig. 2B) and CAP-induced current rises as well as increased Ca^2+^ influxes (Figs. 1–3) are very similar to those described in other corneal cell types [4, 16, 23]. In addition, its invariant expression irrespective of cell passage number also supports the relevance of this cell model. Increased Ca^2+^ influx upon raising the temperature above 43 °C (Fig. 4A) or exposure to 450 mOsmol/l hypertonic stress (Fig. 5A) further indicated functional TRPV1 expression [24, 25]. These responses are pertinent to the in vivo condition since TRPV1 activation is documented to be induced by such stresses and it is alleged to contribute to dry eye symptomology. Specifically, the imposed hypertonic stress is also relevant because it is an established marker used to diagnose dry eye disease [26–28]. It is likely that this imposed stress is pertinent to dry eye induced ocular pain even though in these patients the hyperosmotic threshold for inducing pain through TRPV1 activation is unclear [29].

### L-carnitine suppresses responses linked to TRPV1 activation

Even though L-carnitine is a TRPV1 antagonist in some other ocular cell types [11], such an effect had not been previously described in immortalized HCK cells. Nevertheless, L-carnitine eye drop supplementation in artificial tears helps relieve some dry eye symptomology [30]. Previous to the current study it was only known that L-carnitine acts as an osmoprotectant and inhibitor of MAPK signaling pathway-induced stimulation of proinflammatory cytokine release [7, 10]. Here we show that part of therapeutic effect of artificial tears supplemented with L-carnitine likely stems from its inhibition of TRPV1-induced keratocyte transdifferentiation into myofibroblasts. This conclusion is based on showing that L-carnitine suppressed the following TRPV1-induced responses: (1) 1 mmol/l L-carnitine reduced CAP-induced increases of the whole-cell currents (Fig. 2) and corresponding rises in intracellular Ca^2+^ levels (Fig. 3). (2) L-carnitine reduced both rises in TRPV1 activity induced by hyperosmolarity and a rise in temperature (Figs. 4, 5). Specifically, hyperosmolarity induced by D-mannitol supplementation induced increases in intracellular Ca^2+^ that could be suppressed by 1 mmol/l L-carnitine (Fig. 5B, C). This inhibitory effect is similar to the one described in HCjEC [11]. (3) However, L-carnitine is not such an effective antagonist as CPZ since at a concentration 100-fold higher than CPZ L-carnitine had inhibitory effects on TRPV1 activation that were smaller than those induced by CPZ.

**Fig. 8:**
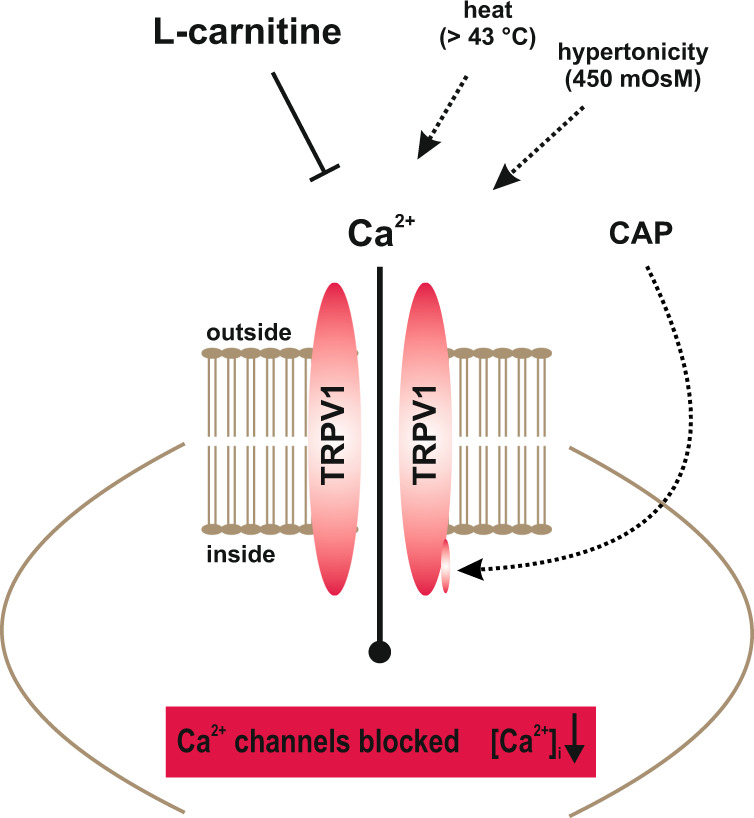
Simplified representation of the effect of L-carnitine on different TRPV1 channel activation pathways. Ca^2+^ channels such as TRPs of the TRPV1 subtype (capsaicin receptor) can be selectively activated by CAP (Figs. 2, 3), heat (>43 °C) (Fig. 4) or hypertonic challenge (Fig. 5) (all dotted arrows). L-carnitine was able to suppress TRPV1 activity at all different TRPV1 activation mechanism (⊥) leading overall to a reduced intracellular Ca^2+^ concentration (Figs. 2–6).

TRPV1 activation by injury contributes to increases in HCK cell migration and suppression of TRPV1 activity by L-carnitine inhibited HCK migration. Similarly, this response also affects control of the HCK phenotype since L-carnitine also reduced keratocyte transdifferentiation into myofibroblasts (Fig. 7). The role of L-carnitine in modulating cell migration was clarified based on showing that L-carnitine already slightly decelerated HCK cell migration over an 8 h period (Fig. 6A–D, E). This inhibitory effect became more pronounced after 24 h (Fig. 6G, J, L). In another study, loss of TRPV1 function impaired healing of an incision stromal wound in mice [3, 31]. This delay was attributed to a lessening of αSMA upregulation, fewer terminally differentiated myofibroblasts delimiting the wound edge and less TGFβ upregulation [3]. These differences may indicate that functional TRPV1 expression contributes to controlling the HCK phenotype since L-carnitine blunted increases in myofibroblast transdifferentiation induced by TGFβ stimulation of TRPV1, albeit to a lesser extent than CPZ (Fig. 7). Our findings support several preliminary studies showing that blocking TRPV1 suppressed myofibroblast formation and expression of TGFβ1 in cultured keratocytes or ocular fibroblasts [31, 32]. The inhibition of TRPV1-driven HCK migration by L-carnitine shown in Fig. 6 is consistent with inhibited wound closure of an incision wound in mice after loss of TRPV1 function [3, 31]. Taken together, L-carnitine is a TRPV1 channel antagonist (Fig. 8) that is less efficacious than CPZ in inhibiting HCK migration and transdifferentiation into myofibroblasts.

### Possible clinical relevance

Loss of corneal transparency is a leading cause of blindness worldwide. Such losses can occur subsequent to either penetrating corneal wounds or stromal infections. Such stresses can increase the conversion of keratocytes and fibroblasts into terminally differentiated myofibroblasts, thereby leading to extracellular matrix remodeling, fibrosis, and opacification. Novel strategies are needed to block these TRPV1 mediated responses induced by TRPV1 activation. The results of the current study suggest that blocking TRPV1 activation on keratocytes with antagonists such as carnitine may prove to be a viable approach to suppress corneal opacification in a clinical setting.

## Data Availability

The datasets used and/or analyzed during the current study are available from the corresponding author on reasonable request.
